# Cholangiocarcinoma Presenting as Uterine Metastasis

**DOI:** 10.1155/2014/204915

**Published:** 2014-12-31

**Authors:** W. Dendas, L. Cappelle, J. Verguts, G. Orye

**Affiliations:** ^1^Department of Obstetrics and Gynecology, Jessa Hospital, Stadsomvaart 11, 3500 Hasselt, Belgium; ^2^Department of Obstetrics and Gynecology, University Hospital KU Leuven Gasthuisberg, Herestraat 49, 3000 Leuven, Belgium; ^3^Department of Pathology, Jessa Hospital, Stadsomvaart 11, 3500 Hasselt, Belgium

## Abstract

Metastases to the female genital tract are rare, with metastatic disease restricted to the uterus being even less frequent. The primary tumor is most often intragenital rather than extragenital. The diagnosis is usually made after occurrence of gynecological symptoms. We describe the case of a 26-year-old female, in whom a curettage for menorrhagia revealed a uterine malignancy, at first thought to be a carcinosarcoma. Biochemistry only showed iron deficiency anemia. Imaging showed discrepant results with liver lesions, suspect of neoplastic or inflammatory disease. She underwent an abdominal hysterectomy and, peroperatively, a frozen section of a mass in the liver hilus demonstrated a cholangiocarcinoma. The diagnosis of a uterine metastasized cholangiocarcinoma was made. We emphasize the fact that uterine metastases have to be excluded in every woman with abnormal uterine bleeding and a personal history of malignancy. However, our case also indicates that gynecological metastatic disease may be the first presentation of an extragenital primary neoplasm.

## 1. Introduction

Metastatic disease is only rarely found in the female genital tract, and it is even more uncommon when the metastases are restricted to the uterus. The primary tumor is more often localized in another gynecological organ rather than oritinating extragenitally [1]. Uterine metastases are most frequently diagnosed after the development of gynecologic symptoms, in casu abnormal bleeding. Patients will often present with a personal history of a malignancy, but the uterine metastasis may also be the first sign of an extragenital malignancy [2].

Gallbladder carcinoma is not only a rare but also a highly lethal disease [3]. Prognosis is poor due to the generally advanced stage at the time of diagnosis [3, 4].

We report a case of cholangiocarcinoma presenting as uterine metastasis.

## 2. Case Report

A 26-year-old G4P2A2 Caucasian woman consulted our emergency department after being diagnosed with a uterine malignancy in Turkey after curettage for menorrhagia. Histopathological examination was suspect for a carcinosarcoma.

Besides obesity, clinical examination was normal. Her appearance was normal without any jaundice. There was no hepatosplenomegaly, nor any lymphadenopathies. In addition, palpation of the breasts showed no abnormalities. A proper gynecological exam was not possible because extreme vaginismus hindered vaginal examination. Vaginal ultrasound showed an enlarged uterus with a normal aspect of the myometrium and a regular delineated endometrium.

Tumor marker CA-125 showed a value of 13,31 kU/L (<35 kU/L). Further biochemical exploration only showed an iron deficiency anemia and slightly elevated Gamma-GT. Other biochemical values are shown in Table 1.

**Figure 1:**
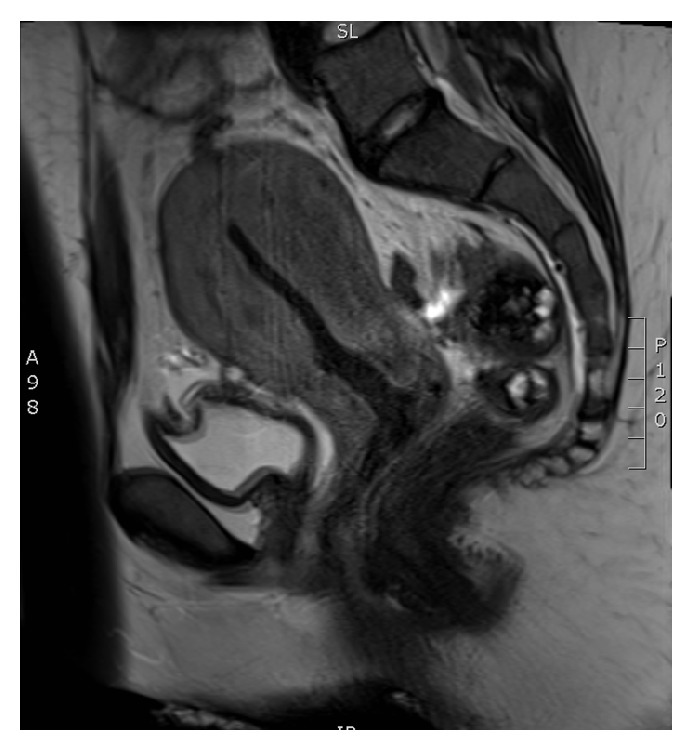
Pelvic MRI showing a large uterus with disappearance of its zonal anatomy, yet lacking any signs of an endometrial tumor.

Abdominal CT showed a heterogeneous uterus, suspect of myometrial or endometrial malignancy, three liver masses, suspect for metastases, an intraperitoneal nodule, suspect for a peritoneal implant, and an irregular thickening of the gallbladder wall, suspect for adenomyomatosis. In contrast, further investigation with pelvic MRI showed a large uterus with disappearance of its zonal anatomy, yet no signs of an endometrial tumor were observed (Figure 1). Furthermore, liver imaging with ultrasound and MRI showed a calculous cholecystitis of the gallbladder fundus and cholangitis with small liver abscesses. Our patient was treated accordingly with intravenous antibiotics. Ongoing menorrhagia was thought to be hormonal and treated with tranexamic acid, progestogens, and repeated blood transfusion.

**Figure 2:**
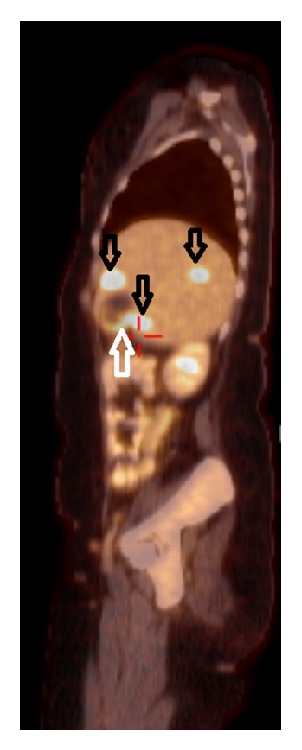
PET-CT showing a hypermetabolic aspect of the uterus (not in this image), gallbladder fundus (white arrow pointing upward), and three liver lesions (black arrows pointing downward), all suspect for neoplastic or inflammatory lesions.

Because of unconfirmed initial pathological results and discrepant imaging results, a PET-CT was performed, which showed a hypermetabolic aspect of the uterus, gallbladder fundus, and three liver lesions, all suspect for neoplastic or inflammatory lesions (Figure 2).

A CT-guided puncture of one liver lesion revealed changes suggestive of a chronic biliary disease, such as primary biliary cirrhosis or primary sclerosing cholangitis, but no malignant cells. Surgery was planned as a new endometrial curettage confirmed the presence of a high grade invasive adenocarcinoma.

Vaginal bimanual examination under anesthesia revealed a nodular aspect of the anterior vaginal wall, although inspection was negative. Furthermore, the cervix, sacrouterine ligaments, and parametria felt indurated. After median laparotomy, a peritoneal nodule was observed against the lower abdominal wall as well as an indurated enlarged uterus with white serosal plaques. Liver palpation revealed an indurated nodule of three centimeters, suspect of a metastasis. Inspection of the upper abdomen showed a suspect irregular white mass in the liver hilus, of which a biopsy was taken. Frozen section showed invasion with a moderately differentiated adenocarcinoma, cytomorphologically comparable to the endometrial biopsy. Because of metastatic disease and history of ongoing uterine bleeding, surgical treatment was restricted to a simple hysterectomy. The ovaries were left in situ.

**Figure 3:**
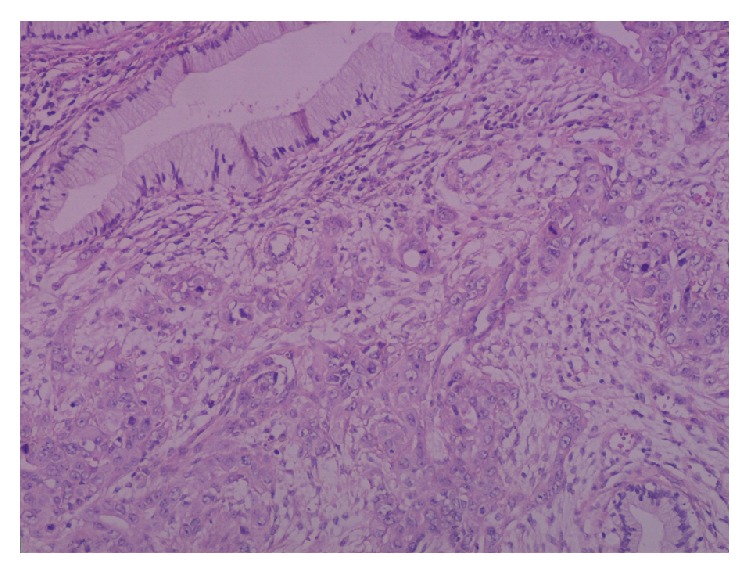
Metastatic cervical wall infiltration, surrounded by normal endocervical glands.

**Figure 4:**
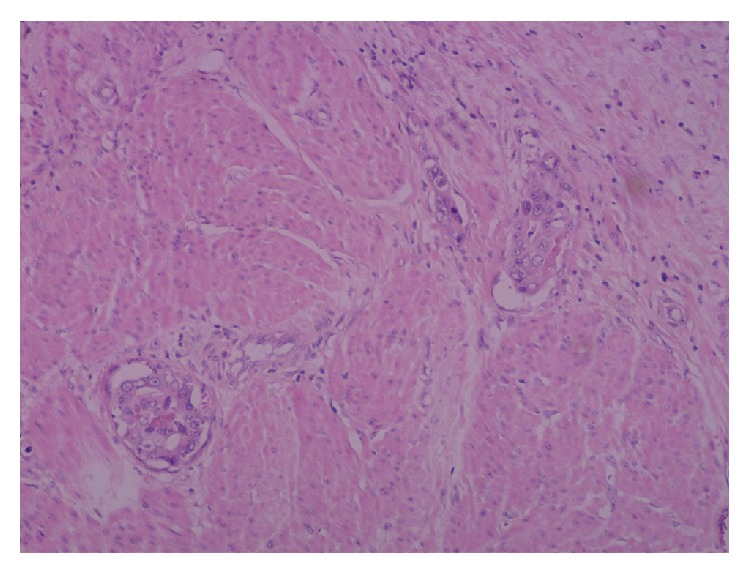
Irregular neoplastic glands infiltrating between the myometrial smooth muscle bundles.

Macroscopic pathological analysis showed a symmetrical enlarged uterus without a localised tumor mass. However, microscopical analysis showed that myometrium and endocervix were diffusely infiltrated by a moderately differentiated adenocarcinoma with obvious desmoplastic stromal reaction. The malignant cells were surrounded by normal endocervical glands in the cervix (Figure 3) and by smooth muscle bundles in the myometrium (Figure 4), a pattern characteristic of their metastatic nature. Immunohistochemical analysis of the gallbladder biopsy was positive for CK19, CK17, CK7, villin, and CEA-p apically. Negative results were obtained for vimentin, calretinin, WT1, neuroendocrine cell markers, ER, and PR. This pattern was compatible with a primary biliary neoplasm. Immunohistochemical analysis of the uterine tumor was compatible with a metastasis of the primary biliary neoplasm. In addition, the peritoneal implant showed an identical image.

Subsequently, our patient with peritoneal-, liver-, and uterine metastasized cholangiocarcinoma received palliative chemotherapy, consisting of cisplatin-gemcitabine.

## 3. Discussion

Metastases to the female genital tract are rare. When occurring, the primary site is most often intragenital [1]. When metastases from extragenital primaries occur, the genital sites affected the most are the ovaries, accounting for 75,8% [1]. Metastatic localisation in the uterine corpus accounts for less than 10%. Kumar and Hart [5] showed that the myometrium was affected by the metastatic disease in 96,2% of their cases, with concomitant endometrial metastases in 32,7%. Uterine metastatic disease was confined to the endometrium in only 3,8% of the patients. Concurrent metastatic disease in the uterine cervix was found in 40,6% and in the ovaries in 65%. Extragenital primary tumors most frequently metastasising to the uterine corpus are summarised in Table 2. These percentages most likely reflect the prevalence of these extragenital cancers in women, without any extragenital cancer having a predisposition to metastasize to the uterus or other organs of the female genital tract [6]. Nevertheless, lobular carcinoma [7] of the breast metastasises more frequently to the female genital tract than ductal carcinoma [8, 9], accounting for 80% [9] of all genital tract breast cancers metastases, although it accounts for only 5–20% of all breast cancers [10].

Stemmermann [11] suggested that uterine metastases are secondary to local lymphatic spread from preceding ovarian metastases and secondary to hematogenous spread when isolated uterine metastases are found [9, 11].

Most often the uterine metastases are diagnosed after the development of gynecologic symptoms, generally abnormal bleeding, with a personal history of a previous primary tumor [2]. Less frequently, the gynecological symptoms are the presenting symptom of an extragenital primary tumor.

Abnormal bleeding occurs when the endometrium is involved [9]. When the metastatic disease is limited to the myometrium, patients may be asymptomatic [9].

Histopathologically, it is clear to tell the metastatic nature of the malignant cells in endometrial sampling, since they infiltrate the stroma without affecting the endometrial glands [2, 7].

Cholangiocarcinoma and gallbladder carcinoma are scarce, with an age standardised incidence rate of 0,5 per 100.000 person-years for gallbladder carcinoma and 1,0 per 100.000 person-years for biliary tract cancer for females in Belgium in 2011 [12]. The number of cases of gallbladder carcinoma metastasising to the uterus is limited, being only 8 to date. They are summarised in Table 3.

Gallbladder carcinoma is not only a rare but also a highly lethal disease [3]. Prognosis is poor due to the generally advanced stage at the time of diagnosis [3, 4], caused by a lack of specific clinical symptoms or signs [3]. Statistics show an overall 5-year survival rate of 5% [3].

Risk factors for development of gallbladder carcinoma are summarised as follows [3]:

 cholecystolithiasis,
 obesity,
 porcelain gallbladder,
 anomalous pancreatobiliary duct junction,
 estrogens (exogenous and endogenous),
 segmental adenomyomatosis of the gallbladder,
 infection,
 carcinogen exposure,
 family tendency.

Chronic inflammation often plays an important role. It is more frequent in women. Furthermore, incidence increases with age, being more frequent in the sixth and seventh decades of life [3], reflecting a progressive evolution from dysplasia to carcinoma in situ and invasive carcinoma, in about 15 years.

Our patient was female and obese, had cholecystolithiasis, had a recent pregnancy, and showed histopathological signs of chronic biliary pathology (type primary biliary cirrhosis/primary sclerosing cholangitis) upon liver biopsy. Nevertheless, our patient was extremely young to develop a cholangiocarcinoma, especially metastatic disease.

Gallbladder carcinoma can spread by different routes, including lymph node spread, direct invasion to the adjacent liver or blood vessels, intraperitoneal spread, neural and intraductal spread, and hematogenous metastasis [3].

In conclusion, metastases to the female genital tract are rare, with disease restricted to the uterus being even less frequent. Nevertheless, uterine metastasis has to be excluded in every woman with abnormal uterine bleeding and a personal history of malignancy [2]. However, gynecological metastatic disease may be established before the diagnosis of an extragenital primary neoplasm.

## Figures and Tables

**Table 1:** Biochemical values at time of admission.

Test	Value	Units	Reference range
Hemoglobin	9,4	g/dL	12,0–16,0
White blood cell count	11,3	×10³/mm³	3,5–11,0
Platelets	727	×10³/mm³	150–400
Iron	24	μg/dL	41–132
Ferritin	3,8	μg/L	11,0–307,0
PT (%)	73,8	%	80,0–120,0
APTT	29,4	sec	25,0–40,0
Creatinine	0,69	mg/dL	0,44–1,03
GFR MDRD	>60	mL/min/1,73 m²	>60
Bilirubin total	0,76	mg/dL	0–1,00
AST (SGOT)	17	U/L	13–42
ALT (SGPT)	17	U/L	10–40
Gamma-GT	49	U/L	5–24
Lipase	23	U/L	22–51
TSH	1,42	mU/L	0,37–3,51
CRP	44	mg/L	0,0–6,0
HCG	<0,5	U/L	<0,5

**Table 2:** Extragenital primary tumors most frequently metastasising to the uterine corpus.

Extragenital site	% [5]
Breast [10]	42,9
Colon	17,5
Stomach [13]	11,1
Pancreas	11,1
Gallbladder	4,8
Lung	4,8
Cutaneous melanoma [14]	3,2
Urinary bladder	3,2
Thyroid	1,6
Hepatocellular carcinoma [15, 16]	Rare
Sarcoma	Rare

**Table 3:** Cases of gallbladder carcinoma metastasising to the uterus.

Article	Number of cases
Charache (1941) [17]	1
Kumar and Hart (1982) [5]	3
Schust et al. (1994) [18]	2 (1 restricted to the cervix)
Martínez-Román et al. (2005) [19]	1 (restricted to the cervix)
Kefeli et al. (2009) [20]	1
